# Web-Based, Open-Source LGBTQ+ Affirming Care Education for Primary Health Care Providers: Descriptive Analysis

**DOI:** 10.2196/92355

**Published:** 2026-09-04

**Authors:** Saniya Sahasrabudhe, Ebony Toussaint, Justin Herrera, Ellesse-Roselee Akré

**Affiliations:** 1 Department of Health Policy and Management, Johns Hopkins Bloomberg School of Public Health, 615 N Wolfe Street, Baltimore, MD, 21205, United States, 1 2134401095; 2 Center for Natural Sciences of Mathematics and Nursing, Bowie State University, Bowie, MD, United States; 3 Department of Population and Family Health, Columbia University Mailman School of Public Health, New York, NY, United States

**Keywords:** lesbian, gay, bisexual, transgender, queer, and other individuals with diverse sexual orientation and gender identities, LGBTQ+, primary health care, culturally competent care, affirming care, gray literature

## Abstract

**Background:**

Affirming care for lesbian, gay, bisexual, transgender, queer, and other individuals with diverse sexual orientations and gender identities (LGBTQ+) populations refers to culturally and clinically competent health care that recognizes specific health needs and provides respectful, inclusive, equitable, and nondiscriminatory services that are supportive of diverse identities. LGBTQ+ populations face greater discrimination in health care, leading to higher levels of unmet health needs than the general population. Very few primary care practices in the United States have training for staff and clinicians on LGBTQ+ health care needs. Despite the growing need for LGBTQ+ affirming care, there are no national standards or requirements for LGBTQ+ cultural competence training for primary health care providers in the United States.

**Objective:**

This study explores the accessibility and quality of online “gray literature” providing LGBTQ+ affirming and culturally competent care information for primary health care providers in the United States. Gray literature is produced by government, academic, business, and industry sources in formats not controlled by commercial publishing.

**Methods:**

We conducted a Google search of the gray literature to identify readily available resources and training materials in January 2024. A total of 2000 websites were screened using inclusion and exclusion criteria. Those published in a language other than English before January 1, 2014, as well as those that were peer-reviewed literature, or behind a paywall, were excluded. Fifty-four websites met the inclusion criteria for a full-text review.

**Results:**

We identified six themes from the existing academic literature: (1) affirming physical and visual environments; (2) sexual orientation and gender identity (SOGI) data collections; (3) training on LGBTQ+ health needs; (4) antidiscrimination policies; (5) appropriate, relevant services for LGBTQ+ patients; and (6) use of inclusive language. We then applied these themes as a deductive coding framework to the web-based sources, and during analysis, two additional emerging themes were identified: (1) staff diversity and (2) health inequalities and inequities. Findings revealed that not every web-based source addressed all themes. This unequal distribution of coverage across these themes means that primary health care providers must consult multiple web-based sources to obtain a comprehensive understanding. Additionally, existing gray literature resources often lacked depth, technical detail, and practical guidance, making it difficult for primary health care providers to access actionable information on LGBTQ+ affirming care. “Training on LGBTQ+ health needs” was the most frequently covered theme, and “SOGI data collection” was the least addressed. Study limitations included geolocation biases and embedded advertisements in the Google search results.

**Conclusions:**

The study highlights that the gray literature is insufficient for self-guided training. We recommend integrating formal LGBTQ+ affirming care training into medical and nursing curricula, as well as professional associations and continuing education, particularly amid growing federal and state-level restrictions on LGBTQ+ health care.

## Introduction

Affirming care is defined as patient-centered medical care that treats each patient with equity, dignity, and respect. It “honors a patient’s identity, acknowledges its impact on the patient’s care needs, and integrates this understanding into the patient’s individual care plan” [1]. It is rooted in the tenets of clinical and cultural competent care [2-4]. Affirming care for lesbian, gay, bisexual, transgender, queer, and other individuals with diverse sexual orientation and gender identities (LGBTQ+) refers to health care practices that recognize specific health needs and provide respectful, inclusive, equitable, and nondiscriminatory services that are supportive of diverse identities [4-6]. While there is no standard definition of LGBTQ+ affirming care, the existing literature indicates six major constructs across interpersonal, institutional, and structural levels: (1) affirming physical and visual environments, (2) sexual orientation and gender identity (SOGI) data collections, (3) training on LGBTQ+ health needs, (4) antidiscrimination policies, (5) appropriate relevant services for LGBTQ+ patients, and (6) use of inclusive language [4,6-13]. Access to LGBTQ+ affirming care and health care providers has been associated with improved management of physical and mental health conditions among LGBTQ+ individuals [4,5]. Studies show greater use of preventive services such as colorectal cancer screening and influenza vaccination, as well as better management of mental health needs when affirming care is available [4].

Compared to cisgender straight persons, members of the LGBTQ+ community experience higher rates of discrimination when accessing health care [14]. One in 3 transgender individuals had to teach their physician about transgender health to receive appropriate care [15]. According to the National Health Care Fairness Survey, nearly 8% of lesbian, gay, and bisexual respondents and 27% of transgender and gender-nonconforming respondents reported being denied needed health care [16]. A study by the Center for American Progress reported that 15% of LGBTQ+ people in the United States reported postponing or avoiding medical treatment due to discrimination. According to minority stress theory [17-19], these negative experiences contribute to the health inequalities demonstrated in the LGBTQ+ community [18-20]. This highlights the need for LGBTQ+ affirming care.

The National Academy of Sciences, Engineering, and Medicine asserts that primary care–based interventions are the key to reducing health inequalities in populations that are vulnerable to being underserved [21-23]. A recent study found that only 34% of primary care practices in the United States provide LGBTQ+ health care training for staff and only 37% provide such training for clinicians [24]. Despite the growing need for LGBTQ+ affirming care, there are no national standards or requirements for LGBTQ+ cultural competence training for primary health care providers in the United States [13,25,26]. There are no standardized requirements for medical school curricula on LGBTQ+ affirming care [27]. A 2017-2018 study by the Association of American Medical Colleges found that while 76% of responding schools included some LGBTQ+ themes in their education, half reported 3 or fewer learning activities, often limited to a single day [27]. Washington, DC, is the only US jurisdiction that mandates physicians complete 2 hours of LGBTQ+ cultural competency continuing medical education (CME) per licensing cycle. Other states, such as California, Nevada, Illinois, Oregon, and New Jersey, require health care providers to undertake broader cultural competency and inclusivity training, which may include LGBTQ+ care as part of the curriculum, but do not specifically mandate LGBTQ+-focused CME [28,29].

In the absence of formal LGBTQ+ affirming care training, health care students and health care providers increasingly rely on point-of-care information resources (UpToDate, Medscape, etc) [30,31], training programs such as TransECHO [32], and open-source online materials (gray literature) to guide practice [31]. Given limited peer-reviewed literature and growing restrictions on LGBTQ+ health care research, the gray literature serves as a rapidly evolving, easily accessible source of current knowledge [31].

Gray literature is increasingly shaping patient and health care provider education and influencing clinical decision-making [31,33-35]. It has gained importance in health care research through its inclusion in systematic reviews [34,36,37] and the growing use of AI-based large language models trained primarily on open-source gray literature [38-40]. The purpose of this study is to describe the availability and quality of web-based gray literature and open-source educational materials on LGBTQ+ affirming health care that are intended to improve patient care in the United States.

## Methods

To understand the types, availability, and quality of open-source resources available to primary health care providers, we conducted a descriptive analysis. We selected a descriptive analysis instead of a systematic review to highlight the availability of resources *outside* peer-reviewed journals and paid training sessions. To identify readily available resources and training materials, we searched the gray literature, including websites, blogs, and policy documents. We analyzed these web sources (websites) for best practices and information that primary health care providers could implement in their workflows to provide LGBTQ+ affirming and competent care.

### Ethical Considerations

This study was exempt from institutional review board approval and informed consent because it involved a descriptive analysis of publicly available gray literature and did not involve human participants or the collection of primary data.

### Search Strategy

We searched the gray literature using Google in January 2024. We narrowed our search to 10 search terms that included (but were not limited to) “lgbt” OR ”gender nonconforming” OR “trans” OR “gay” OR “queer” AND “affirming” AND “primary care,” “inclusive AND healthcare,” “supportive AND care,” “friendly AND care,” “inclusive” AND “healthcare” AND “recommendations,” “physician AND “provider”AND “training,” and “staff” AND “training.”

### Eligibility

We included websites, blogs, policy documents, and reports that outlined standards, recommendations, or suggested practices for delivering and improving LGBTQ+ affirming health care in various types of primary health care settings. Materials were limited to those relevant to health care in the United States and excluded those intended specifically for patient education or caregivers. We included sources specific to primary health care practices, while excluding web-based sources that focused exclusively on mental health, palliative care, or nursing home care. We included sources that addressed primary care for LGBTQ+ patients. If the sources were addressed to caregivers, parents, and families of the LGBTQ+ patients, they were excluded. Details of the eligibility criteria are illustrated in Table 1.

**Table 1.** Eligibility criteria for web-based, open-source lesbian, gay, bisexual, transgender, queer, and other individuals with diverse sexual orientation and gender identities (LGBTQ+) affirming care educational web sources for health care providers included in the descriptive analysis.

	Inclusion criteria	Exclusion criteria
Population	LGBTQ+ individuals	Family of LGBTQ+ individuals or non–LGBTQ+ individuals
Exposure	Primary care in physical health care settings	Before January 1, 2014
Comparator	—^a^	Non-English language and non–United States
Outcomes	LGBTQ+ affirming and culturally competent care	Not a source of standards, recommendations, and suggested practices for providing competent care
Study design	Open-source gray literature	Peer-reviewed literature

a Not applicable.

### Primary Outcomes

Our study team identified that LGBTQ+ affirming care has been understood by researchers at large, but there is no standard definition. The study team conducted a preliminary literature review of existing academic literature on various aspects of LGBTQ+ affirming health care [4,6-13]. We identified six common themes across interpersonal, institutional, and structural levels, as listed in Table 2: (1) affirming physical and visual environments, (2) SOGI data collections, (3) training on LGBTQ+ health needs, (4) antidiscrimination policies, (5) appropriate relevant services for LGBTQ+ patients, and (6) use of inclusive language.

**Figure 1.**
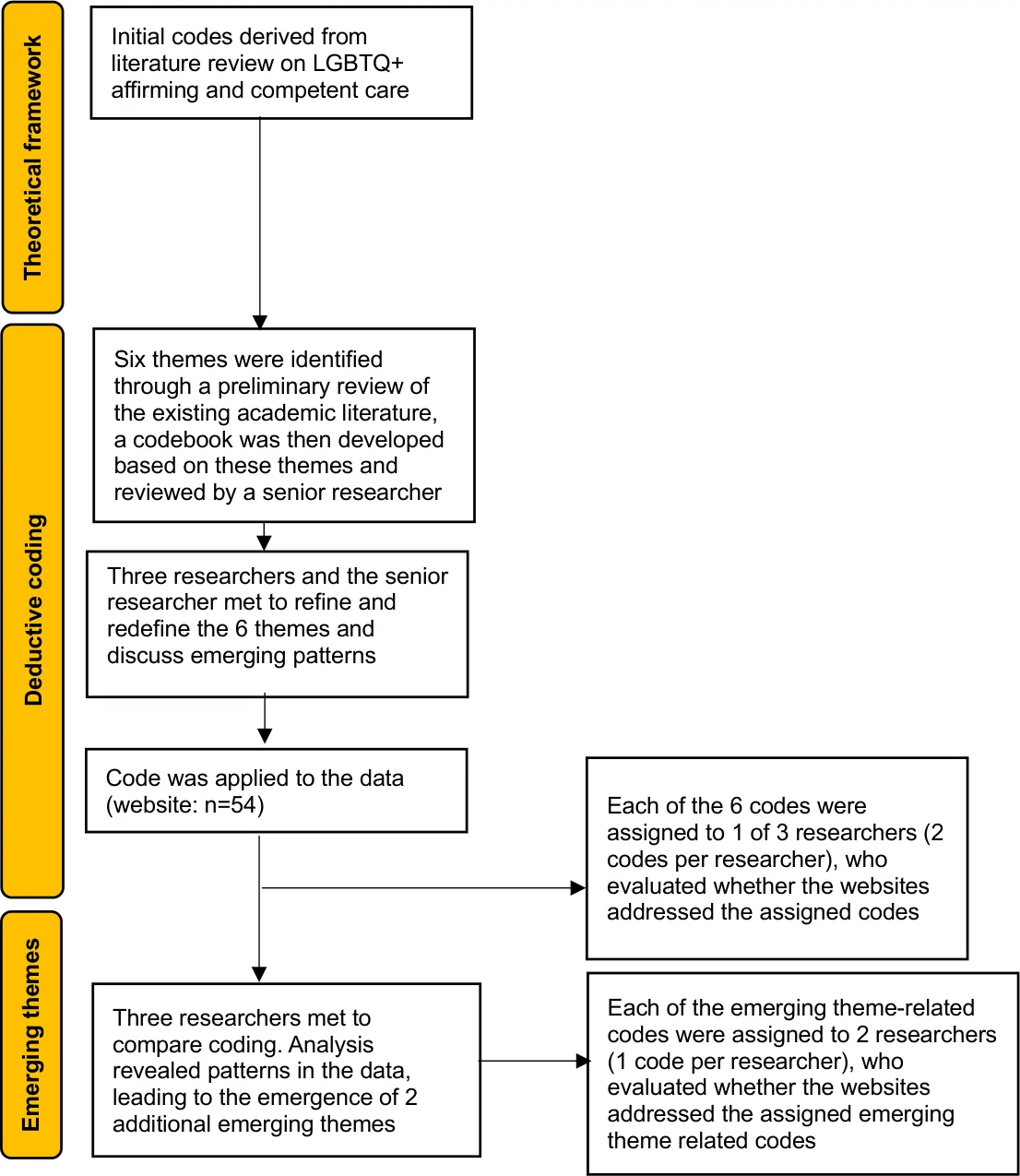
Flow diagram of the deductive thematic analysis applied to 54 web sources as part of a descriptive analysis, depicting how preidentified themes were applied and how emerging patterns were identified and categorized into emerging themes. LGBTQ+: lesbian, gay, bisexual, transgender, queer, and other individuals with diverse sexual orientation and gender identities.

Using these preidentified themes, we conducted deductive coding of the web-based sources, and through the analysis, researchers identified 2 additional emerging themes, as shown in Table 2 and Figure 1 [41-43]. Each code was assigned to 1 of 3 researchers, who evaluated whether the websites addressed the assigned codes. To ensure intercoder reliability, 1 coder cross-checked at the end of the coding process whether the assigned codes were appropriately addressed and present on the websites.

**Table 2.** Number of open-source web sources addressing lesbian, gay, bisexual, transgender, queer, and other individuals with diverse sexual orientation and gender identities (LGBTQ+) affirming care, organized by theme (preidentified and emerging).

Themes addressing LGBTQ+ affirming care	Websites that address a theme n (%)
Preidentified themes (for deductive thematic analysis)	
Affirming physical and visual environments	35 (64.8)
Sexual orientation and gender identity data collections	19 (35.2)
Training on LGBTQ+ health needs	40 (74.1)
Antidiscrimination policy	32 (59.3)
Appropriate and relevant services for LGBTQ+ patients	23 (42.6)
The use of inclusive language	47 (87)
Emerging themes (through inductive thematic analysis)	
Staff diversity	9 (16.7)
Health inequalities and inequities	32 (59.3)

### Screening and Data Extraction

Two researchers searched 10 terms each and retrieved 2,798,860,000 results from the Google search engine. Out of these, the first 100 search results for each term were identified. From these 2000 searches, duplicate weblinks (n=1041) were identified and excluded. The remaining 959 websites were independently screened by 3 reviewers based on site title, description, and a short text review, with disagreements resolved through consensus. We excluded websites (n=638) published before January 1, 2014, unrelated to the United States, or not providing standard recommendations or suggested practices for LGBTQ+ competent care. The full text of 321 websites was reviewed for eligibility by 3 reviewers, and 267 websites that did not meet the inclusion criteria were excluded. Finally, 54 websites that met our eligibility criteria were included in our review. Three researchers independently reviewed the websites and reached a consensus that these 54 websites provided accessible and high-quality information meeting key criteria for credible health information sources, and conflicts were resolved through comprehensive discussion. For the websites included in the analysis, researchers were able to clearly identify who runs them (academic organization, governmental agency or department, or nonprofit organization, etc) and the purpose of the website. Finally, websites sponsored by the health care businesses and practices themselves were excluded. An example of a quality source is the “Creating an Inclusive Environment for LGBT Patients” forms and policy brief developed by the National LGBT Health Education Center at Fenway Health [44]. A complete list of included websites can be found in the Multimedia Appendix 1.

**Figure 2.**
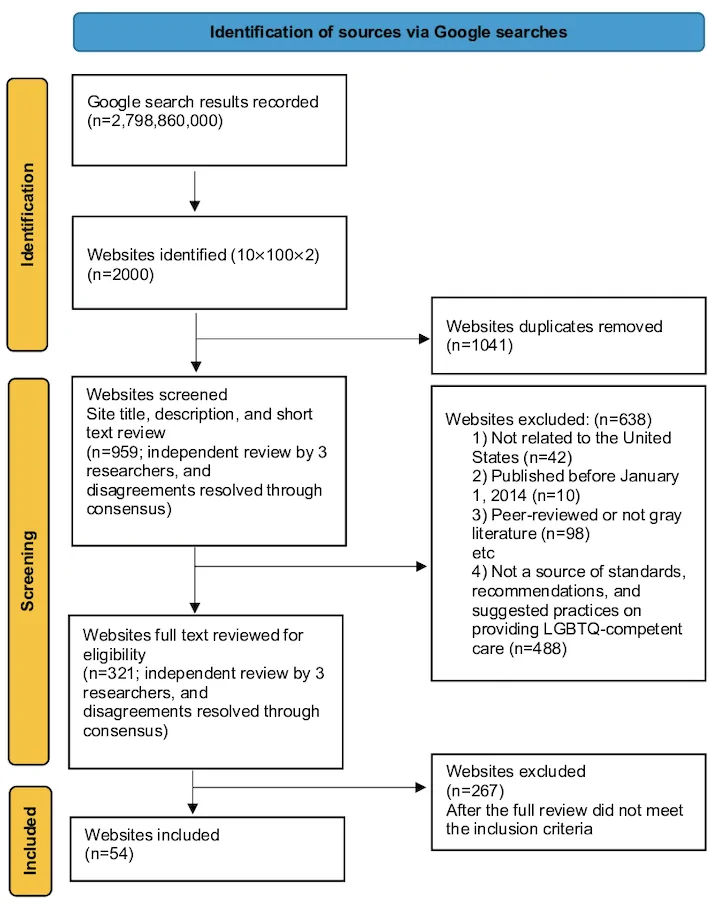
PRISMA (Preferred Reporting Items for Systematic Reviews and Meta-Analyses) flow diagram of the selection process for open-source gray literature, focusing on educational resources on lesbian, gay, bisexual, transgender, queer, and other individuals with diverse sexual orientation and gender identities (LGBTQ+) affirming health care for health care providers.

The process is illustrated in Figure 2.

## Results

### Overview

Six themes of LGBTQ+ affirming health care were identified through a literature review. A full-text review of the 54 sources was conducted to determine if they addressed any of the 6 identified themes using deductive coding, as shown in Figure 1 and Table 2 illustrates the number of sources that addressed each theme. Web-based sources often addressed multiple themes concurrently. Our short text review of the 959 web-based sources identified 29 websites behind a paywall.

### Affirming Physical and Visual Environment

Among the 54 sources analyzed, 35 addressed the theme of creating an affirming and welcoming physical and visual environment. Most of these sources emphasized the importance of displaying pride flags; pronouns on identification badges; inclusive marketing; and health educational materials such as magazines, pamphlets, brochures, and office signage. Inclusive patient intake forms and prominently displayed nondiscriminatory policies, which highlight the process for sharing feedback and complaints, are also critical components.

While antidiscriminatory policies were frequently mentioned as displayed visibly, we did not identify guiding examples of optimal policies. One source proposed considering patient flow through the primary care office and providing visual cues at every step, from the front desk to the exam room, to facilitate voluntary information sharing and privacy. Additionally, the importance of having a diverse staff was highlighted.

Eighteen web-based sources mentioned the importance of inclusive restrooms, with 3 specifically highlighting “all-gender single occupancy” restrooms. None of the sources, however, detailed the budget or infrastructure requirements for these inclusive changes.

### SOGI Data Collection

Nineteen of 54 websites emphasized the importance of collecting, documenting, and avoiding assumptions about patients’ SOGI during primary care visits. It typically focused on proactively collecting these data in the demographic section of the clinical interview, with a focus on 3 questions: 1 question about sexual orientation, 1 about sex assigned at birth, and 1 about current gender identity.

### Antidiscrimination Policies

Thirty-two web-based sources were categorized as “antidiscrimination policy.” These sources featured practical advice, implementation examples, and a checklist for an organization to evaluate its inclusivity efforts. The web-based sources that we reviewed suggested adding LGBTQ+ people to the organization’s existing nondiscrimination policy to ensure LGBTQ+ inclusivity and that all staff should be knowledgeable of the hospital’s nondiscrimination policy. While a few web-based sources recommended incorporating nondiscrimination language addressing “sexual orientation,” “gender identity,” “HIV status,” and “gender expression,” others provided general recommendations to promote inclusivity of LGBTQ+ patients.

Some sources recommended that hospitals and practices add an equal visitation policy or a written nondiscrimination policy for LGBTQ+ families. Six web-based sources provided examples of antidiscriminatory policies, many of which linked to external examples from organizations such as the Department of Health and Human Services, Centers for Medicare and Medicaid Services, The Joint Commission, American Medical Association, Funders for LGBTQ+ Issues, and the Human Rights Campaign.

Six sources suggested that hospitals should provide a clear process for LGBTQ+ patients and their families to report discrimination if it occurs during their appointments, but did not provide clear instructions on the reporting process. While some resources emphasized having a nondiscrimination policy to inform practice, others suggested having a visible policy on the website or in the office for patients to see and feel more welcome. These suggestions overlapped with “affirming physical and/or visual environment.”

### Training on LGBTQ+ Health Needs

Forty resources were categorized as “training on LGBTQ+ health needs.” Several sources encouraged LGBTQ+ affirming care staff training as part of the Human Rights Campaign’s Health Equality Index objectives.

The sources that targeted health care providers commonly included training focused on LGBTQ+ health disparities or awareness training, guides defining LGBTQ+ terms for physicians, and data collection practices. The full-text review of the web-based sources emphasized the importance of training staff on “gender inclusive” practices, “how to use pronouns,” “gender-affirming care,” and “LGBTQ+ patient care” to improve their professional competence. Some sources suggested implementing an annual LGBTQ+ cultural competency training for all staff and requiring all new employees to attend primary training before beginning work. This could help mitigate negative experiences caused by inadequately trained personnel, thereby improving patient comfort and trust.

Many resources suggested providing certificates or CME credits for health care providers who voluntarily participated in the training. Most sources did not provide substantive content on what should be included in the training.

### Appropriate, Relevant Services

The sources under this theme primarily focused on the services needed by the LGBTQ+ patient population, emphasizing essential services such as hormone replacement therapy, HIV and sexually transmitted infection (STI) screenings, and birth control options. However, many sources lacked detailed guidance for health care providers on important primary care services for transgender and gender-nonconforming patients. Specifically, there was a lack of information on cancer risks and screenings; tailored safe sex advice; immunizations; and screenings based on the dimensions of the patient’s sexual orientation (eg, behavior or attraction), sex assigned at birth, and gender identity.

The mention of reproductive health care for LGBTQ+ patients often focuses heavily on STI prevention, neglecting family planning and fertility services that are affirming to all families [45].

### Inclusive Language 

The “inclusive language” theme encompassed 47 sources, making it the most addressed theme. A common recommendation across these sources was the inclusion of preferred pronouns on registration forms to facilitate respectful communication. Sources provided detailed guidance on prioritizing gender-neutral language in interactions, offering insights into effectively serving patients with diverse gender identities during appointments. However, few sources provided practical demonstrations of interactions with patients; guidance on conducting organ inventory; or advice on referring to anatomy, such as using “chest” instead of “breast” [45]

Resources most commonly provided terminology glossaries with definitions of terms such as “transgender,” “gender-nonconforming,” “bisexual,” and “asexual.” Comparatively fewer resources addressed outdated terms such as “hermaphrodite,” “sexual preference,” and “sex change,” and very few went on to suggest the preferred alternatives such as “intersex,” “sexual orientation,” and “gender-affirming surgery” [45]. Resources that did not include terminology glossaries tended to briefly mention the importance of using correct pronouns without further elaboration on navigating nuanced situations and handling mistakes.

In addition to the 6 themes discussed, our full-text review revealed recurring discussions on health inequities (often called health disparities) and staff diversity. Consequently, we categorized these observations into *2 independent emerging themes*.

### Health Inequalities and Inequities

Of the 54 reviewed sources, 32 included a section called “health disparities” or something similar, which included information on health outcome inequities. They create a contextual argument for inclusive health care [46]. These sources provided statistical data on the negative experiences faced by LGBTQ+ patients, such as higher rates of mental health issues, delayed medical care due to fear of discrimination, and lower overall satisfaction with health care services. The sources presented these statistics to underscore the critical need for inclusive, culturally competent, patient-centered care.

### Staff Diversity

Nine resources highlighted the importance of having LGBTQ+ staff in primary care offices. These articles stress that a diverse workforce fosters a respectful, inclusive environment and demonstrates an organization’s commitment to LGBTQ+ equity.

One resource suggested adding inclusive language to job applications to encourage LGBTQ+ applicants and posting job listings on diverse LGBTQ+ periodicals and websites. Web-based sources also discussed the importance of retention of LGBTQ+ employees. They called for equity-promoting protocols such as parental and caregiving leave, creating LGBTQ+ affinity groups, and offering comprehensive health insurance plans that cover LGBTQ-specific needs. These practices can help create a comfortable workplace. One article linked the treatment of LGBTQ+ staff to patient care, stating that visible LGBTQ+ staff members make diverse patients feel more welcome.

## Discussion

### Principal Results and Comparison With Prior Work

This study evaluated the availability and quality of the gray literature and open-source LGBTQ+ health education materials online. Our findings reveal a significant lack of organized, easily accessible gray literature for self-education and training. Much of the available content is obscured by layers of advertisements, LGBTQ+ glossaries, and redundant information.

In this study, we understand that accessing quality information through a Google search is inefficient. Motivated health care providers looking for information for self-education or training would have to scan through multiple sources before they could identify concrete information related to LGBTQ+ competent care. We reached this understanding by reviewing the 2000 web sources identified through our search. Of these, 1041 were duplicates, and only 54 were deemed relevant for a full-text review (Figure 2). Consequently, only 2.7% (54/2000) of the search results offered content specifically addressing LGBTQ+ affirming or culturally competent care in primary care settings. The descriptive details provided by these sources were generally limited.

Each theme represents an essential component for a holistic understanding of LGBTQ+ affirming care (Table 2). As reflected in the results, not every web-based source addressed all themes. This unequal distribution of coverage across these themes means that health care providers must consult multiple web-based sources to obtain a comprehensive understanding. If health care providers do not carefully select a diverse range of sources, relying solely on the top search results of the gray literature could leave significant gaps in their knowledge about the breadth and scope of the LGBTQ+ affirming care.

“Training on LGBTQ+ health needs” was the most common theme, appearing in 40 of the 54 web-based sources reviewed. “SOGI data collection” was the least addressed theme; only 19 web-based sources addressed this theme. Only 9 gray literature sources addressed the importance of the diversity and representation of LGBTQ+ people in the primary health care provider’s office. Thirty-three web-based sources addressed health outcome inequities, often under the title of health disparities. We observe that health disparity framing does not necessarily address structural components, such as race, gender, income, and built environment, that contribute to health stress [46,47].

Additionally, in the full-text analysis of the 54 resources, we observed a significant lack of technical details and concrete illustrated examples. Providing descriptive details such as budget or infrastructure requirements for the inclusive changes could motivate decision-makers to implement LGBTQ+ affirming care practices. This lack of technical and descriptive detail fails to motivate or effectively improve health care provider competencies, perpetuating barriers for the LGBTQ+ community in accessing comprehensive, informed, and compassionate care.

The frequency and content of topics addressed on the websites we reviewed align with those reported in peer-reviewed research. Most websites reviewed in our study addressed LGBTQ+ affirming care interventions at the “interpersonal” level, such as using inclusive language and creating a welcoming environment. Comparatively, very few websites addressed interventions at the “institutional” level, such as implementing equal visitation and nondiscrimination policies and hiring and retaining staff with diverse identities. The reviewed websites rarely addressed interventions at the “structural” level to advance LGBTQ+ affirming care, such as increasing insurance coverage and improving affordability of health care [4].

In our findings, SOGI data collection was the least addressed theme. Asking 3 key questions—1 about sexual orientation, 1 about sex assigned at birth, and 1 about current gender identity—can help establish trust, reduce unconscious biases, and create an affirming environment for discussions between the health care provider and patient. The addition of these 3 key questions can lead to better patient-centered care [12,48].

Integrating the collection of SOGI data alongside information on the social determinants of health into standard clinical interviews can help address health inequities and improve patient-centered care [12]. Having 3 SOGI questions kept separate from sexual history questions during clinical interviews has been better received by patients, as sexual history questions often feel irrelevant or intrusive in primary care visits that are not related to sexual or reproductive health [44,48,49]. Moreover, SOGI data collection is an affirming component of primary care, helping to create supportive check-in opportunities.

Additionally, it is imperative to note the importance of staff training in collecting SOGI data in an affirming manner and ensuring that SOGI data are interoperable across different electronic medical record systems. Without interoperability and appropriate staff training to collect SOGI data, patients may need to repeatedly provide this information when receiving care from multiple health systems.

In our search and screening, we found no resources that encouraged primary health care providers to initiate and continue follow-up for hormone therapy or discuss family planning, fertility preservation, and reproductive treatment options essential for providing LGBTQ+ competent care. This lack of guidance often forces LGBTQ+ patients to seek specialists for care and guidance that heterosexual patients can typically receive from their primary health care providers [24,50].

On the basis of our analysis, the consistent use of inclusive language stems from broader efforts within primary care practices to establish a strong antidiscriminatory culture and a clear process of having diverse, representative, and well-trained staff. This finding indicates that, while we describe each of the 6 themes independently, they are interconnected.

Given the gaps in current health care provider training, continued medical education, and existing peer-reviewed literature focused on LGBTQ+ affirming care, this study focused on investigating the nature and quality of alternative open-source gray literature available to educate primary health care providers on improving the quality of LGBTQ+ affirming care. Future studies that systematize a conceptual framework for LGBTQ+ affirming health care are needed to support further health services research, clinical applicability, and implementation of activities related to LGBTQ+ affirming care across various physical and mental health care settings.

Of the 54 web sources identified and analyzed in the study, 41 remain accessible as of April 2026, highlighting the evolving nature of information available through the internet and search engines. Notably, certain academic, government, and military websites have since become inactive, probably due to the evolving policy and political landscape.

### Limitations

We encountered 2 notable limitations in our analysis. First, we encountered geolocation issues, where our search results were influenced by our location, leading to information about health care facilities specific to Maryland [51]. These location-specific results were excluded from our short text analysis. Given Maryland’s progressive politics, it is likely that search results could differ in more conservative states. While our goal was to analyze data in a nationally relevant way, the geolocation-influenced search results may have limited our reach. Second, information was difficult to access because of the embedded advertisements. Multiple Google advertisements required extensive scrolling on the web page to reach the actual content of the articles. Additionally, many sources encountered were health care provider or practice advertisements that encouraged patients to seek service at a specific facility rather than offering tools for health professionals to deliver LGBTQ+ affirming care. Given the objectives and design of this study, along with its inclusion and exclusion criteria, the results and analysis are not extended to peer-reviewed academic literature, non-English literature, or paywalled gray literature and web sources. Furthermore, given the limited technical details available in the gray literature web sources, the findings and analysis do not comment on the ease of applicability and implementation of LGBTQ+ affirming care.

### Conclusions

On the basis of this study, which reflects the lack of quality and detail in gray literature sources, it is difficult for primary health care providers and practice-level decision-makers to independently pursue further education in LGBTQ+ affirming and competent care. Gray literature or cursory web-based training cannot suffice the need for comprehensive and formal instructional training. To increase the prevalence of confident and competent LGBTQ+ affirming health care providers, formal clinical training in LGBTQ+ affirming care during medical education is essential. For health care providers who have already completed their professional training, we recommend that state licensing bodies consider offering regular courses and training on LGBTQ+ affirming care as part of the CME. Beyond clinical competency and interpersonal cultural competency, these trainings could focus on implementing effective and sustainable clinical practice–level changes to address the health needs of LGBTQ+ patients, thereby driving change at the institutional level.

Finally, despite the political targeting of LGBTQ+ health research, it is crucial to support and promote health services research that furthers the understanding and implementation of LGBTQ+ affirming care, generates robust evidence to help refute hateful and harmful rhetoric, and improves the health care experiences and health outcomes of LGBTQ+ individuals.

## Supplementary material

10.2196/92355 Multimedia Appendix 1 List of websites analyzed after full-text screening and the themes of lesbian, gay, bisexual, transgender, queer, and other individuals with diverse sexual orientations and gender identities affirming care comprehensively addressed in each.

## Abbreviations

CME: continuing medical education
LGBTQ+: lesbian, gay, bisexual, transgender, queer, and other individuals with diverse sexual orientations and gender identities
SOGI: sexual orientation and gender identity
STI: sexually transmitted infections
